# Diagnostic test accuracy for COVID-19: systematic review and meta-analysis protocol.

**DOI:** 10.23889/ijpds.v7i3.1818

**Published:** 2022-08-25

**Authors:** Helen Daniels, Rhiannon K Owen

**Affiliations:** 1 Swansea University

## Objective

Accurate diagnosis of COVID-19 infection is paramount to initiating appropriate measures for reducing spread. We aim to conduct a systematic review and meta-analysis, augmented by linked electronic health records, to assess the diagnostic test accuracy for COVID-19.

## Approach

We will search the following databases from November 2019 to February 2022: MEDLINE (PubMed), Embase, and Scopus, as well as reference lists of eligible studies and review articles. Keywords will relate to COVID-19 and diagnostic testing.

Eligible studies will use an appropriate study design (e.g. prospective and retrospective cohort and case-control) to assess the accuracy of any COVID-19 diagnostic test (including thoracic imaging, mass spectrometry, and serological tests) in all healthcare and community settings. Studies of participants under 18 will be excluded.

Data will be extracted using a piloted extraction form and bias will be assessed using the QUADAS-2 tool.

## Results

Main outcomes will include frequency statistics, sensitivity and specificity, and positive and negative predictive value. Paired forest plots will be used to illustrate sensitivity and specificity across studies. We will pool data on sensitivity and specificity in a Bayesian framework using a bivariate random-effects logistic regression model, where appropriate. Uncertainty in the estimates will be represented using 95% credible intervals.

A comparative framework will be developed to allow assessment of the comparative accuracy of diagnostic tests. Subgroup analyses will be undertaken for time since onset of symptoms, setting (including community and secondary care testing), and reference standard, where appropriate.

## Conclusion

Results of this review will be combined with routinely collected electronic health records from the DECOVID database to inform relationships between tests and subgroups for healthcare decision-making. New methodology developed as part of this review will be generalizable to the evaluation of diagnostic test accuracy in other diseases.

